# Supplementary material for: Heterogeneous Structural Disturbance of Cell Membrane by Peptides with Modulated Hydrophobic Properties
Source: Pharmaceutics. 2022 Nov 15;14(11):2471. doi: 10.3390/pharmaceutics14112471 (PMC9692774; doi:10.3390/pharmaceutics14112471)
Supplement: Supplementary file 1 [file pharmaceutics-14-02471-s001.zip › pharmaceutics-2025372-supplementary.pdf]

Supporting Information for

Heterogeneous structural disturbance of cell membrane by peptides with modulated hydrophobic properties

A

	Amino acid sequence																		
As-CATH4	1	2	3	4	5	6	7	8	9	10	11	12	13	14	15	16	17	18	19
	R	R	G	L	F	K	K	L	R	R	K	I	K	K	G	F	K	K	I
	20	21	22	23	24	25	26	27	28	29	30	31	32	33	34	35	36	37	38
	F	K	R	L	P	P	V	G	V	G	V	S	I	P	L	A	G	R	R

B

	Amino acid sequence																
	1	2	3	4	5	6	7	8	9	10	11	12	13	14	15	16	17
AS4	R	G	L	F	K	K	L	R	R	K	I	K	K	G	F	K	K
AS4-1	R	G	L	F	K	K	L	L	R	K	I	K	K	G	F	K	K
AS4-5	L	G	L	F	K	K	L	L	R	L	I	K	K	G	F	K	K
AS4-9	L	G	L	F	K	K	L	L	R	L	I	L	K	G	F	K	L

Figure S1. Amino acid sequences of the natural peptide As-CATH4 (A), the amino acid 2-18 fragment AS4, and the variants AS4-1, AS4-5, and AS4-9 (B).

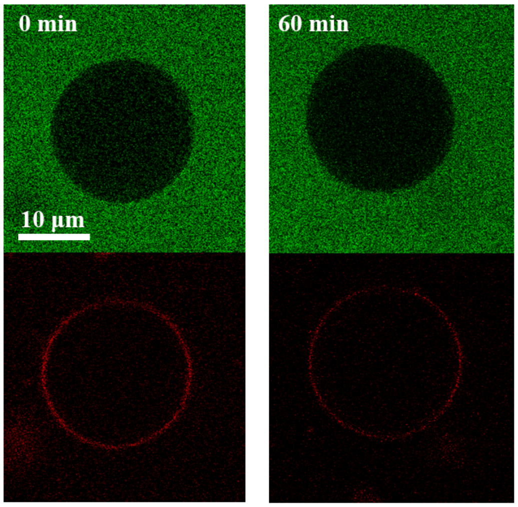

Figure S2. Confocal images of a GUV dispersed in calcein solution without peptide addition. The GUV keeps stable without calcein entry under observation.

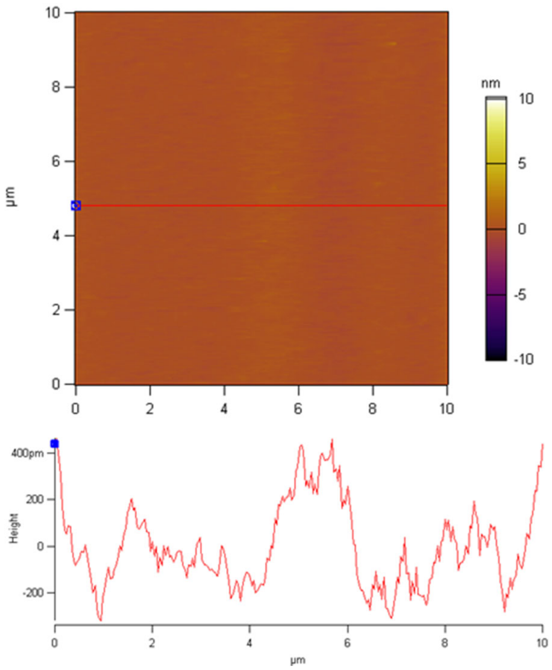

Figure S3. AFM image of a pristine SLB before peptide exposure. The orange background refers to SLB surface. Corresponding height profile is shown at the bottom.
